# Pulsatile lavage irrigator tip, a rare radiolucent retained foreign body in the pelvis: a case report

**DOI:** 10.1186/1754-9493-5-14

**Published:** 2011-05-28

**Authors:** Camille L Connelly, Michael T Archdeacon

**Affiliations:** 1 Department of Orthopaedic Surgery, University of Cincinnati, P.O. Box 670212, Cincinnati, OH, 45267-0212, USA

**Keywords:** retained foreign body, radiolucent, pulsatile lavage, surgical complication, patient safety

## Abstract

Retained foreign bodies after surgery have the potential to cause serious medical complications for patients and bring fourth serious medico-legal consequences for surgeons and hospitals. Standard operating room protocols have been adopted to reduce the occurrence of the most common retained foreign bodies. Despite these precautions, radiolucent objects and uncounted components/pieces of instruments are at risk to be retained in the surgical wound. We report the unusual case of a retained plastic pulsatile lavage irrigator tip in the surgical wound during acetabulum fracture fixation, which was subsequently identified on routine postoperative computed tomography. Revision surgery was required in order to remove the retained object, and the patient had no further complications.

## Background

Retained foreign bodies are rare but serious events in patient safety. There is abundant surgical literature regarding the most common retained foreign bodies: surgical gauze, sponges and metallic instruments [1-4]. As a precaution, preoperative and postoperative instrument, sponge and needle counts are standard procedure in the operating suite. Additionally, metallic threading in surgical sponges and routine intraoperative and postoperative imaging are safeguards to prevent retained objects in the surgical wound. However, pieces of instruments that break-off or come apart unnoticed are at risk to be retained in the wound. Furthermore, retained radiolucent objects are not detectable on plain radiographs and may escape detection if patients do not become symptomatic or if advanced imaging is not obtained. We report a case involving a retained pulsatile lavage irrigator tip in the surgical wound after acetabulum fracture fixation.

## Case Presentation

A sixty-four year old man was transferred to our trauma center from an outside hospital after sustaining a left acetabulum fracture in a fall on ice. The patient was stable on admission and complained of severe left hip pain, without loss or change in sensation. Examination revealed no gross hip deformity; however, left hip pain was elicited on log roll. A neurovascular exam revealed no deficits preoperatively. No other injuries were detected.

Radiographs and preoperative computed tomography (CT) scan demonstrated a left anterior column, posterior hemitransverse acetabulum fracture [5], OTA 62-B3.2 [6] and an ipsilateral nondisplaced inferior pubic ramus fracture. The patient was placed in balanced skeletal traction in the emergency room. The risks and benefits of surgery, as well as alternative treatments, were discussed with the patient and consent for surgery was obtained. The patient was evaluated by the medicine team and cleared preoperatively.

**Figure 1:**
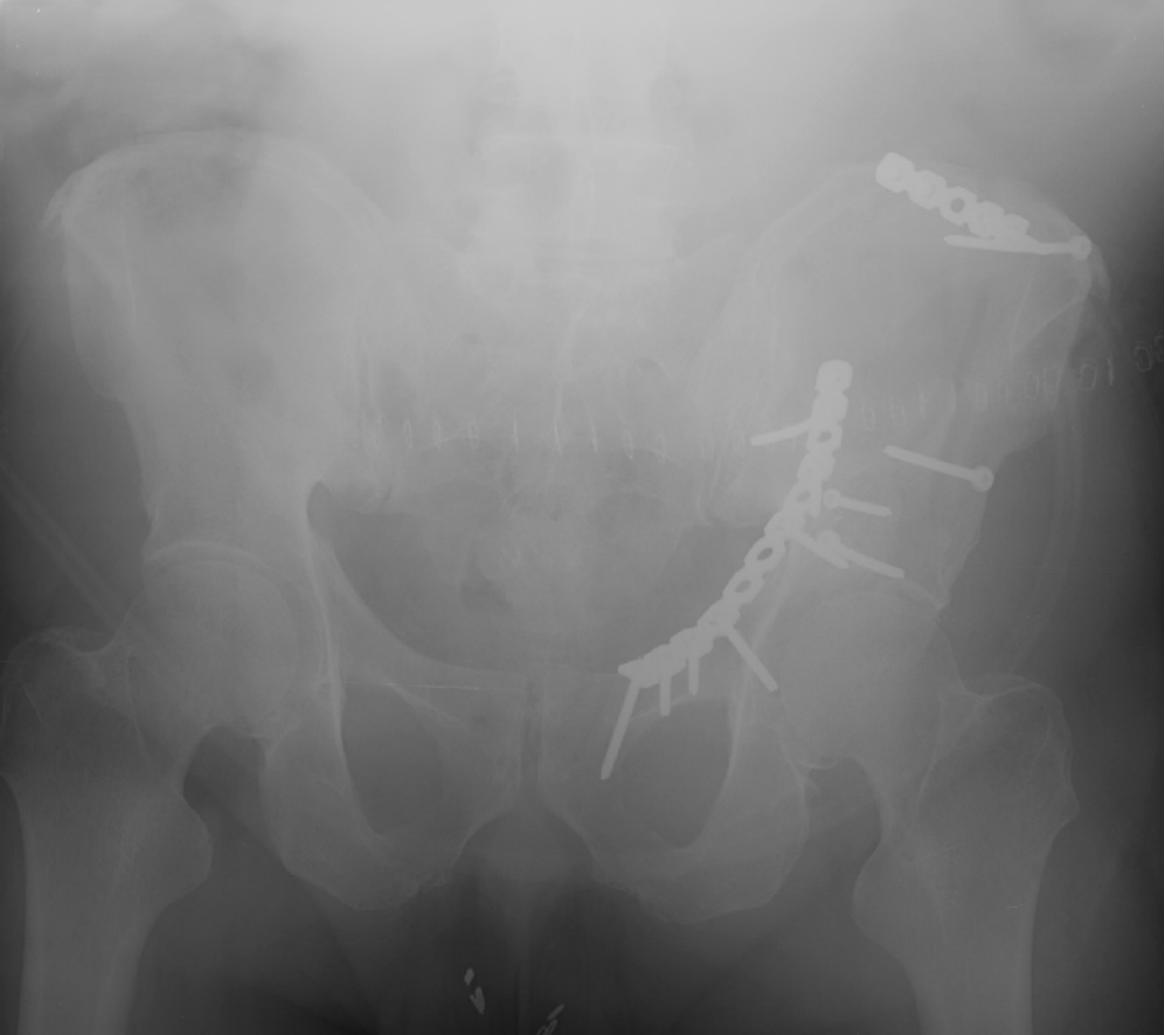
**Immediate postoperative AP and oblique pelvic radiographs demonstrating near anatomic reduction status post ORIF**.

**Figure 2:**
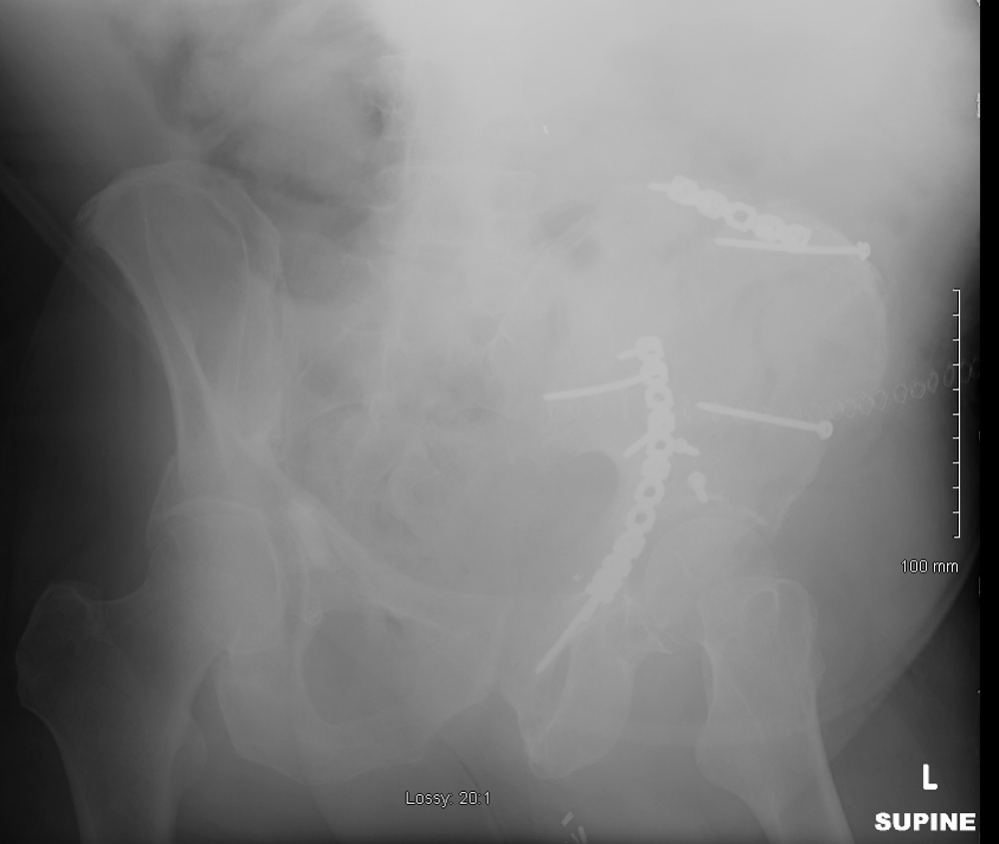
**Immediate postoperative AP and oblique pelvic radiographs demonstrating near anatomic reduction status post ORIF**.

**Figure 3:**
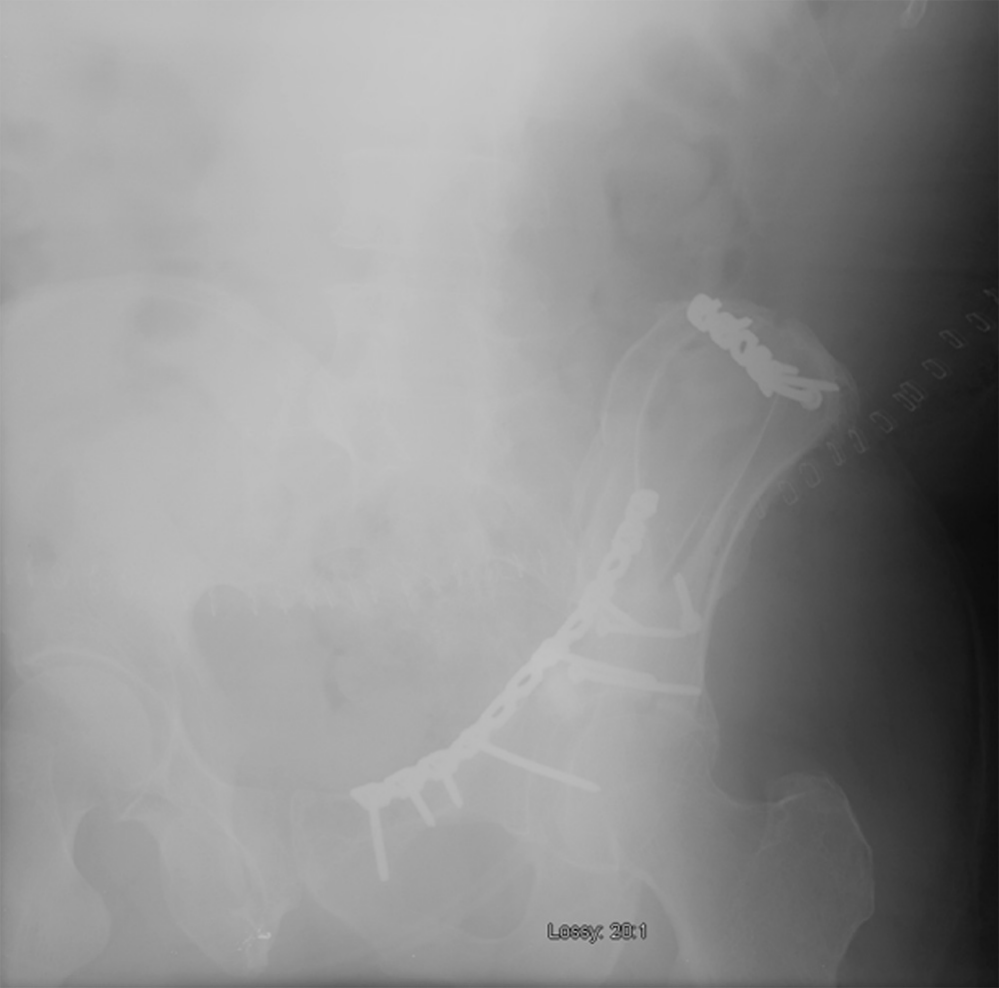
**Immediate postoperative AP and oblique pelvic radiographs demonstrating near anatomic reduction status post ORIF**.

The patient underwent open reduction internal fixation (ORIF) of his acetabulum fracture through a modified ilioinguinal Stoppa approach [7]. The wounds were irrigated with three liters of pulsatile normal saline and closed in layers as is routine at our institution. There were no recognized intraoperative complications and instrument and sponge counts were correct. Immediate postoperative anterior-posterior (AP) and Judet radiographs (Figure 1, Figure 2 and Figure 3) demonstrated near anatomic reduction of the acetabulum, no evidence of intra-articular hardware penetration and a concentric reduction of the hip. The patient was transferred to the recovery room in stable condition.

**Figure 4:**
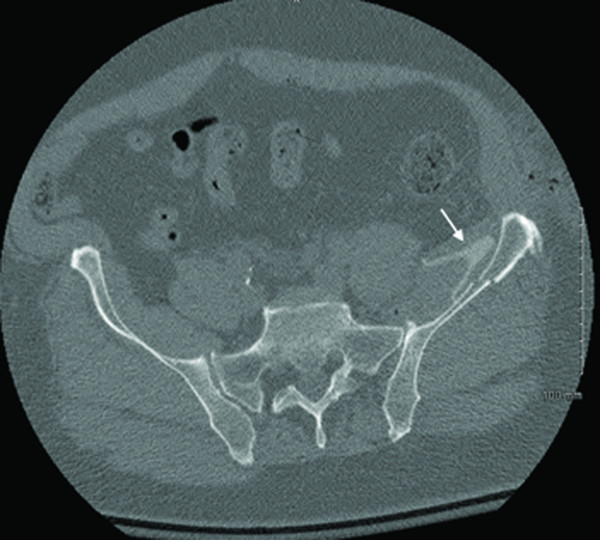
**Postoperative axial and coronal pelvic CT scans showing an irregular, hyperdensity (white arrow) in the left iliacus muscle**.

**Figure 5:**
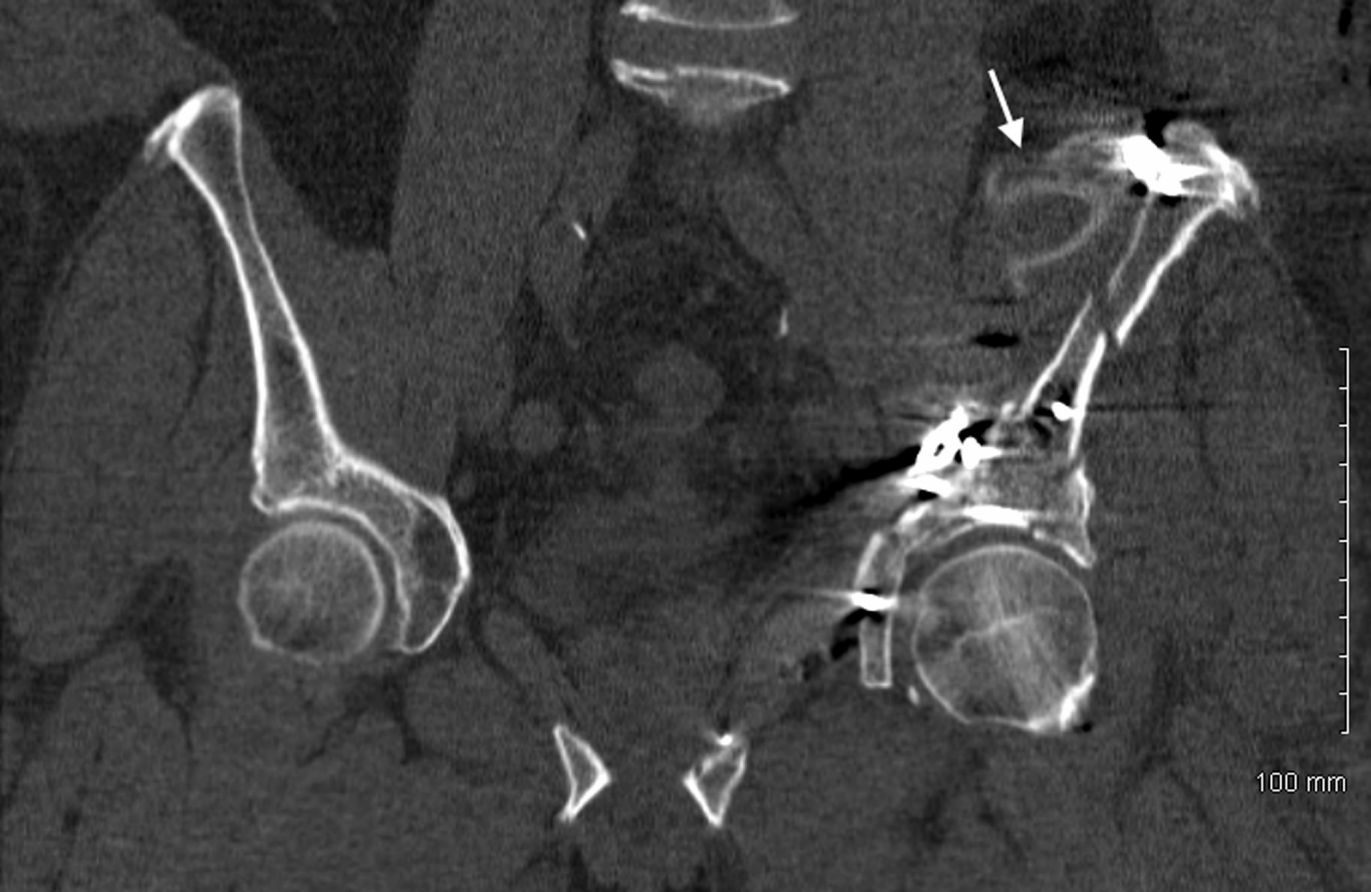
**Postoperative axial and coronal pelvic CT scans showing an irregular, hyperdensity (white arrow) in the left iliacus muscle**.

A standard postoperative CT of the pelvis was obtained as is routine at our institution. This revealed a concentric reduction of the hip joint without intra-articular implant penetration. However, the attending orthopaedic surgeon noted an ipsilateral irregularly shaped hyperdensity in the left iliacus muscle and operative bed. The discrepancy was measured at 250-300 Hounsfield units (HU) and appeared without significant associated streak artifact (Figure 4 and Figure 5, white arrows). It was not present on the preoperative CT scan and was not detectable on any of the preoperative or postoperative plain radiographs. Thus, suspicion was raised for a retained foreign body versus an atypical hematoma. Based on the imaging characteristics, a retained foreign body was favored and exploratory revision surgery was recommended.

**Figure 6:**
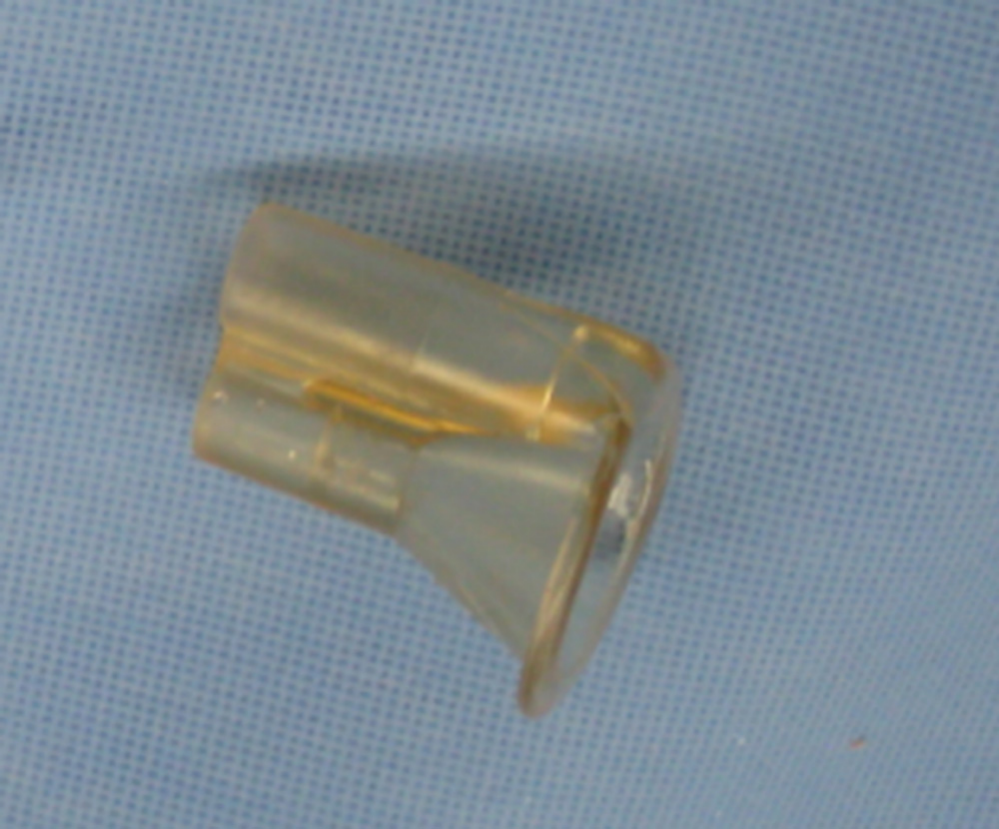
**The retained foreign body was identified as the nozzle tip from the pulsatile lavage irrigation system**.

Upon wound exploration, a retained foreign body was confirmed and identified as the plastic tip from the pulsatile lavage unit that had been used for wound irrigation during the first procedure (Interpulse High Flow Tip model 210-14, Stryker, Kalamazoo, MI) (Figure 6). This was removed without complication. Postoperative neurovascular examinations were intact and unchanged from previous. The patient was discharged on hospital day eight in stable condition.

## Discussion

Non-textile radiolucent retained foreign bodies after surgery have been rarely reported in the literature [3]. Unintentional retained foreign bodies after surgery have the potential to cause serious medical complications for patients and bring fourth serious medico-legal consequences for surgeons and hospitals [1,3,4] and are considered "never events" by the National Quality Forum (NQF) and Centers for Medicare and Medicaid Services (CMS) [8,9]. While strict enforcement of operating room safeguards minimizes the risk of medical errors, the inherent risks of surgery, including the placement of foreign material inside the body, prevents complete elimination of this possibility. It is thought that approximately 1,500 cases of unintentional retained foreign bodies occur in the United States each year [1,2,4].

Although the majority of iatrogenic retained foreign bodies are detected soon after surgery [1,4] others are not detected until many years later [4]. Radiolucent foreign bodies are a particular challenge for detection and require a high index of suspicion. Prevention through instrument inspection and accounting for all radiolucent components used in surgery are the best safeguards to avoid these errors.

Unintentional retained foreign objects after surgery may be asymptomatic or lead to complications including pain, infection, or abscess formation. Occasionally foreign body migration has been noted to result in substantial morbidity [10,11] and even death [12]. Fortunately, the foreign body in this case was removed without further complication.

**Figure 7:**
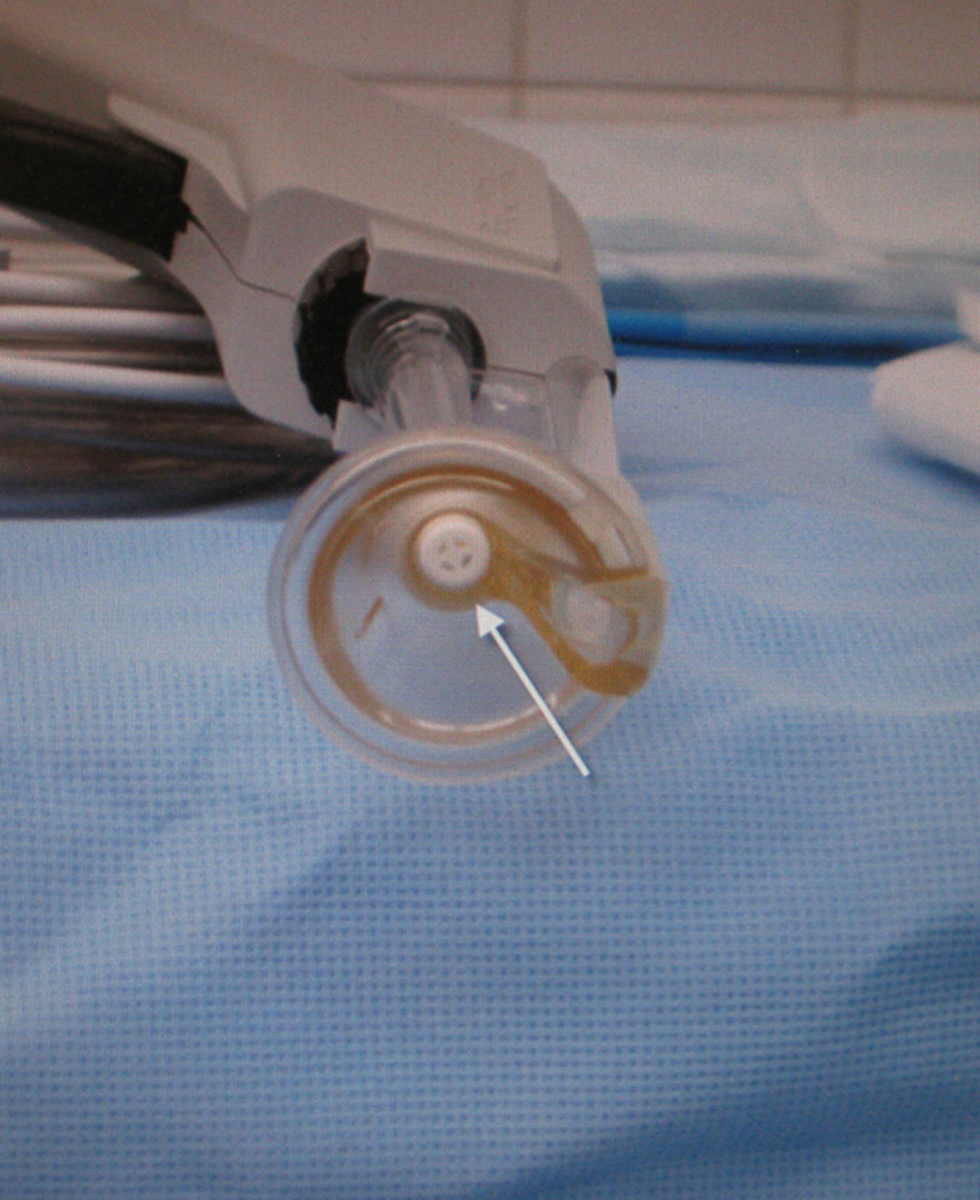
**An end-on view of the pulsatile lavage nozzle with the central filter cap (arrow)**.

**Figure 8:**
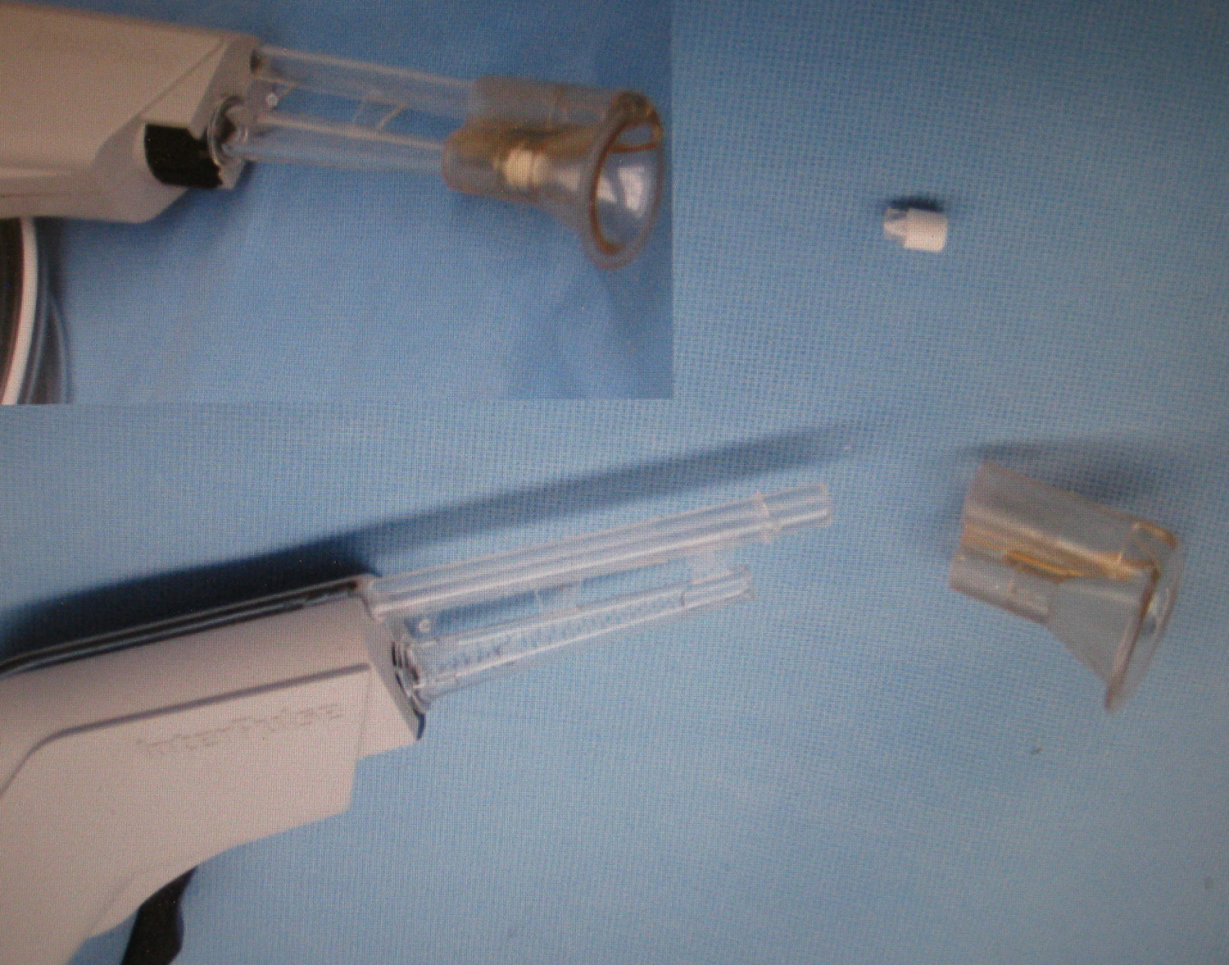
**The pulsatile lavage system with disassembled and assembled (inset) components**.

Following this event, a root cause analysis was performed to determine the precipitating factors, and to prevent recurrence of this complication. The first issue identified was a process-related error involving surgical equipment modification. In the experience with the Interpulse Powered Lavage System (Stryker, Kalamazoo, MI) at our institution, it was perceived that the irrigation time in operating room was longer than desired. It was also noted that by removing the central "filter cap" in the tip of the irrigator (Figure 7 and Figure 8, white arrow) that a higher flow could be achieved, reducing irrigation time. In operating room time trials this difference was determined to be approximately 45 seconds for each 3-liter bag of saline. Thus, it had become standard practice in our operating rooms to remove this component on the back table, prior to use. However, in light of this event, we have discontinued this practice. We suspect that the central filter cap may add some stability to the fixation of the nozzle tip on the lavage apparatus. Thus, removing this piece may have contributed to the dislodgment of the tip within the pelvic wound. Still, we are not aware of any other events or close-calls with a dislodging irrigator nozzle tip at our institution or in the literature.

While we hope that eliminating this practice of instrument modification will prevent any similar events in the future, we have also instituted several other preventive measures. Because the tip was not a recognized risk for dislodgement and becoming a separate piece, it was not individualized as part of the operative count. Therefore, the second identified root cause issue regards adding lavage nozzle tips to the operative count as an early warning. Additionally, thorough wound inspections will be completed with an increased awareness for the risk of retained instrument components/pieces and nozzle tip dislodgement.

Furthermore, a higher level of suspicion for radiolucent retained foreign bodies will be considered. Undetectable on intraoperative and immediate postoperative imaging, retained radiolucent objects may not be discovered unless the patient becomes symptomatic or unless advanced imaging is ordered. In this case, the diagnosis was not suspected until a discrepancy was noticed on the routine postoperative CT scan. Thus, the incompatibility of radiolucent foreign bodies with standard early detection methods contributed to delayed detection and a return to the operative suite.

Finally, there should be heightened awareness for potential retained foreign bodies with surgical procedures involving large body cavities (abdomen, pelvis, chest) [3] or patients with elevated body mass indices (BMI) [1]. This case included both risk factors, a patient with a BMI of 37.5 and large pelvic wound bed.

Although standard operating room counts, wound explorations and careful intraoperative imaging prevent most unintentional retained foreign bodies, radiolucent foreign bodies are a particular challenge for detection and require a high index of suspicion. We present this case to share awareness for potential pulsatile lavage nozzle tip dislodgement and advise that instrument modification may sacrifice connection integrity. We suggest that particular attention should be paid while utilizing instruments or equipment with radiolucent components in surgery and that instrument components should be individually counted items. We reiterate the importance of standard operating room procedures: time-outs, instrument and sponge counts, wound inspection and careful assessment of intraoperative/postoperative imaging.

## Conclusion

Radiolucent retained foreign bodies are not easily detected and there is potential for uncounted components to become unintended retained foreign bodies. When using powered lavage systems we advise against equipment modification. We also advocate the addition of the nozzle tip to the countable items list and recommend thorough inspection and palpation of surgical wounds immediately prior to closure.

## Consent

Written informed consent was obtained from the patient for publication of this case report and any accompanying images. A copy of the written consent is available for review by the Editor-in-Chief of this journal.

## Competing interests

The authors declare that they have no competing interests.

## Authors' contributions

CC was involved in acquisition of the data and drafting of the manuscript. MA made substantial contributions to the design, drafting and final approval of the manuscript. All authors read and approved the final manuscript.
